# Using Hand Grip Force as a Correlate of Longitudinal Acceleration Comfort for Rapid Transit Trains

**DOI:** 10.3390/s150715755

**Published:** 2015-07-02

**Authors:** Beiyuan Guo, Weide Gan, Weining Fang

**Affiliations:** State Key Laboratory of Rail Traffic Control and Safety, Beijing Jiaotong University, No.3 Shang Yuan Cun, Beijing 100044, China; E-Mails: 10121644@bjtu.edu.cn (W.G.); wnfang@bjtu.edu.cn (W.F.)

**Keywords:** hand grip force, longitudinal acceleration comfort, rapid transit train, motion simulation system

## Abstract

Longitudinal acceleration comfort is one of the essential metrics used to evaluate the ride comfort of train. The aim of this study was to investigate the effectiveness of using hand grip force as a correlate of longitudinal acceleration comfort of rapid transit trains. In the paper, a motion simulation system was set up and a two-stage experiment was designed to investigate the role of the grip force on the longitudinal comfort of rapid transit trains. The results of the experiment show that the incremental grip force was linearly correlated with the longitudinal acceleration value, while the incremental grip force had no correlation with the direction of the longitudinal acceleration vector. The results also show that the effects of incremental grip force and acceleration duration on the longitudinal comfort of rapid transit trains were significant. Based on multiple regression analysis, a step function model was established to predict the longitudinal comfort of rapid transit trains using the incremental grip force and the acceleration duration. The feasibility and practicably of the model was verified by a field test. Furthermore, a comparative analysis shows that the motion simulation system and the grip force based model were valid to support the laboratory studies on the longitudinal comfort of rapid transit trains.

## 1. Introduction

As green travel is becoming popular with the public, more and more people are choosing rapid transit systems to travel in metropolis. People have high requirements for riding comfort in rapid transit systems. Riding comfort can be influenced by many factors, such as acceleration performance, interior space [1], noise and vibration [2], ventilation [3], temperature [4], seat design [5] and passenger services [6]. Longitudinal acceleration is one of the essential factors that effects the riding comfort of trains [7]. Longitudinal acceleration comfort has been widely considered in the areas of vehicle design, such as adaptive cruise control design [8] and braking system design [9].

Longitudinal acceleration comfort research has been carried out since the 1930s [10]. So far, some relevant standards, such as TB/T2370 and ISO 2631-1, have been released. The standards stipulate the acceptable maximum acceleration value of the traction and braking system of the train [11], which can be used as the vehicle design basis. However, the value is a minimum requirement for the ever-growing comfort requirements of passengers. Hence, further research was promoted to investigate the longitudinal acceleration comfort of passengers. For example, Feng *et al*. investigated the longitudinal acceleration comfort of passengers in different resting and reading postures [12].

Longitudinal acceleration comfort can be measured by objective or subjective approaches [10]. The subjective approach use questionnaires. Selected participants, who are placed in vehicles or laboratory devices, are asked to write down their sensations about various motions on a questionnaire after being exposed to the motion. Since the sensation a participant describes may not indicate how he behaves, subjective approaches need to recruit enough participants to obtain statistically feasible results. Objective approaches use some physical quantity to directly evaluate the comfort level. An objective approach has the advantage of calculating the result through the single measurement of the physical quantity. The physical quantities recently used to measure longitudinal acceleration comfort include acceleration and balance retention [10]. Most tests use acceleration as the physical quantity, as it can be acquired through accelerometers [13] or the accelerometer in a mobile phone [14].

For security and efficiency reasons, researchers usually prefer using laboratory devices to using real vehicles, for example, Fard discussed the effects of seated structural dynamics on ride comfort criteria using a vibration simulation system [15]. With the development of simulation technology, a motion simulation system now can convey the effect of gravitational forces and cues participants experience during kinematic changes in acceleration. Motion simulation systems also have disadvantages. The motion simulation systems simulate acceleration by tilting forward and backward. It should be noted here that the acceleration sensor can measure the acceleration of the simulation system, but cannot measure the acceleration of the simulated train. Hence, there’s impossible to measure the acceleration of the simulated train directly with an accelerometer.

It is hard for a standing passenger to maintain his balance because the center of his mass is higher than the one of a seated passenger in a rapid transit train. As the train accelerates or decelerates, the passenger must to hold on to a handrail and exert significant forces to maintain his balance. Hence, the changes of the grip force are supposed to be a feasible criterion to calculate the longitudinal acceleration comfort of the passengers. In order to objectively evaluate the longitudinal acceleration comfort of passengers in a real train or on a motion simulation system, the novel idea of using pressure map systems to evaluate the longitudinal acceleration comfort is presented in the this paper.

A pressure map is a system for assessing pressure distribution. The system includes a thin sensor mat which can be placed on a working surface. The system also includes a computer used to process the sensor data. When something is put on the working surface, a map of pressure data will be recorded by the system for subsequent further analysis. Pressure map systems are widely used in the area of clinical medicine [16], product design [17], manufacturing [18], ergonomics [19], sport [20], among others.

In the present study, a motion simulation system was used to simulate the working conditions of a rapid transit train, and a pressure map system was used to record the changes of hand grip force under different working conditions. The goal of the study was to determine the correlation between comfort score and hand grip force to extend the use of the pressure sensors.

## 2. Methodology

### 2.1. Simulation Setup

We built a motion simulation system, which was composed of a motion system, a standing platform and a virtual simulation system, to simulate the motion of a passenger’s standing situation.

#### 2.1.1 Motion System

**Figure 1:**
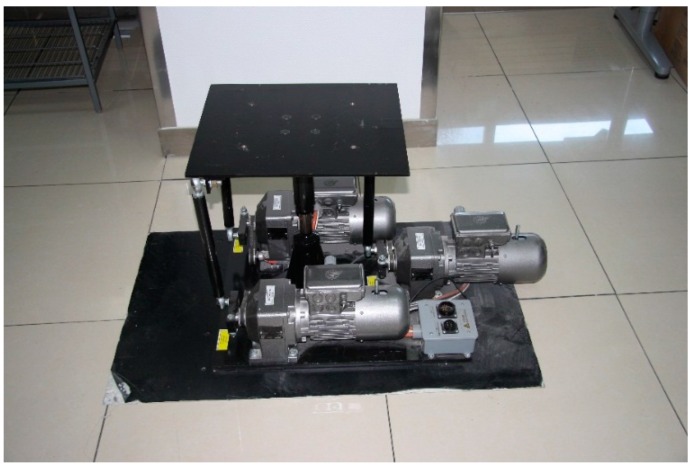
The three axis motion system.

A three axis electric motion system (MSE 710S-3, MSE Simulation Ltd.) was used to generate the pulse. The system (see Figure 1) had three electric motors, which could work together to drive the upper frame of the system to tilt forward and backward, tilt side to side and move up and down. The ranges of the pitching, rolling and heaving motions of the system were −15° to 15°, −15° to 15° and −5 cm to 5 cm, respectively. The response speeds of the pitching, rolling and heaving motion of the system were 60°/s, 60°/s and 33 cm/s, respectively.

The motion system cannot simulate a long acceleration process due to its space limitations. Hence, the constantly acceleration was simulated by tilting participant backwards and using the gravity vector as a replacement for the forward acceleration force. As the tilting could cause the movement along the vertical axis (Z axis), a Z axis compensation based algorithm was designed and used to drive the motion system. The participants’ standing vestibular heights were used as the input parameter of the motion system.

#### 2.1.2. Standing Platform

**Figure 2:**
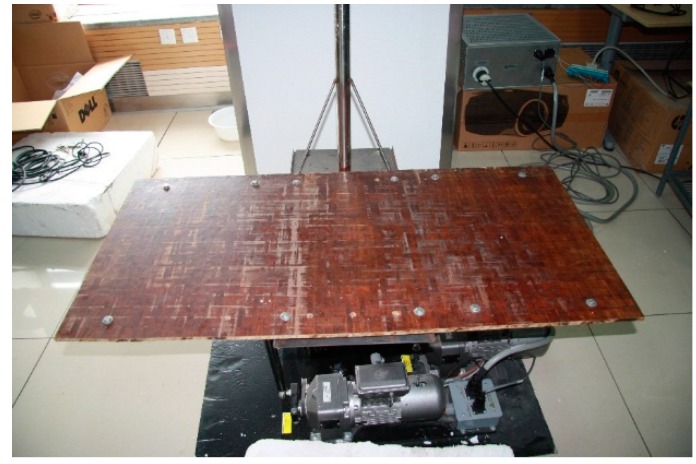
The standing platform setup.

The standing platform was made of three components: a steel frame, a support plate and a stainless steel tube. The steel frame was the load bearing structure of the platform. The support plate was a piece of bamboo plywood that participants could stand on. The length and the width of the plate were 1200 mm and 600 mm, respectively. The stainless steel tube, which was welded on the steel frame as a vertical handrail, was 38 mm in diameter. The diameter was as same as the diameter of real handrail on trains. The plate was screwed onto the steel frame. The standing platform was bolted to the upper frame of the motion system. The standing platform setup is shown in Figure 2.

#### 2.1.3. Virtual Simulation

**Figure 3:**
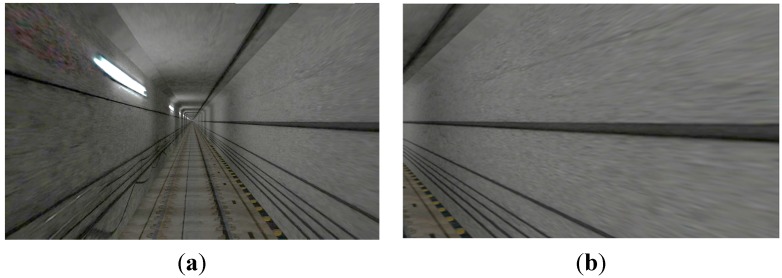
Examples of visual simulation scene: (**a**) Front view; (**b**) Side view.

A virtual simulation system was used to improve the user immersion of simulation scenarios. The virtual simulation system included visual simulation and audio simulation. Computer generated imagery (CGI) was used to create the visual scene. The scene included all the rapid transit system elements, such as tracks and subsidiary facilities of the rapid transit system, and landscapes on both track sides. Examples of the visual simulation scene for the experiment scenarios are shown in Figure 3.

An audio simulation system was used to generate the sound of the train running, including the track noises, braking noises, among others.

**Figure 4:**
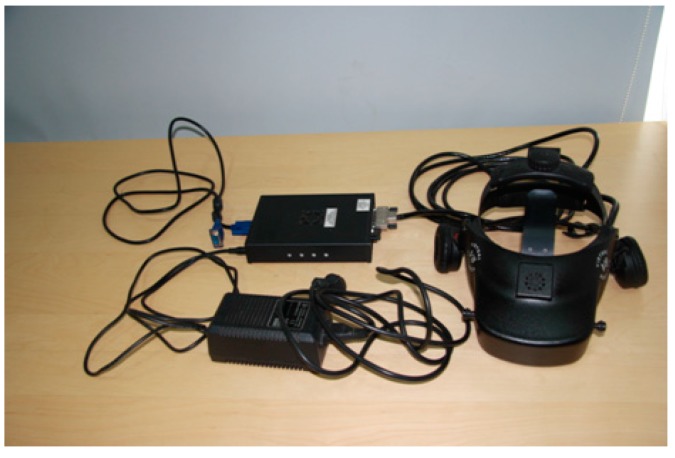
V8 head mount display.

A head mount display (HMD, Virtual Research V8, Aptos, CA, USA) was used as the visual and aural display. The HMD (see Figure 4) had dual 1.3″ diagonal active matrix liquid crystal displays with resolution of 640 pixels in width and 480 pixels in height. The field of view of the HMD was 60° diagonal.

### 2.2. Participants

In total fifteen participants were recruited from students in Beijing Jiaotong University. All participants were male, with an average age of 23.3. The participants were all right-handed. The stature of the participants ranged from 1650 mm to 1870 mm (average stature, 1752 mm. Participants were ask to avoid strenuous exercise or physical activity for 24 h before the experiment, to preclude possible variations in motion sensation. For safety, participants with high blood pressure, heart, back or neck problems, motion sickness were excluded from the study. All the participants had previous experience of taking rapid transit trains. The participants were able to understand the test procedures.

### 2.3. Apparatus

**Figure 5:**
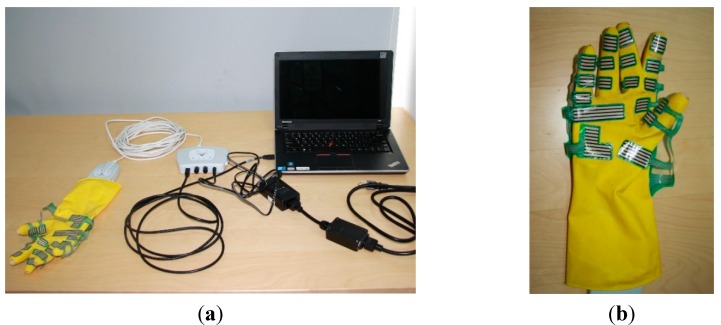
(**a**) Tekscan grip pressure mapping system; (**b**) Sensor mounted on a right hand rubber glove.

A Tekscan (South Boston, MA, USA) grip pressure mapping system (see Figure 5a) was used to gather the hand grip forces of participants. The system was composed of a Tekscan pressure mapping sensor, a VersaTek cuff and a VersaTek hub. The cuff connected to the sensor to gather, process and send data to a computer via the hub. The properties of the sensor (#4256E) are described in Table 1. The scan rate of the system was up to 750 Hz. For convenience, the sensor was stuck on a right hand rubber glove (see Figure 5b).

**Table 1:** Properties of the pressure sensor.

Name	Value
Accuracy	Better than ±10%
Linearity	<±3%
Repeatability	<±3.5%
Hysteresis	<4/5%
Drift per log time	<5%
Lag Time	5 µs
Spatial Resolution	As fine as 0.6 mm × 0.6 mm
Thinness	0.1 mm
Pressure range	0–345 kPa

### 2.4. Experimental Design

We proposed a two-stage experimental designs which allow for early stopping with a negative result of the first-stage experiment.

#### 2.4.1. Stage One

The first-stage experiment evaluated whether the simulation setup was suitable for simulating the longitudinal pulse of a rapid transit train. Six acceleration and deceleration working conditions, which were similar to the working conditions of Beijing metro line 4, were chosen as the experimental working conditions. Table 2 describes the six working conditions in detail.

Before the start of the experiment, a signed informed consent document was obtained from the participants. Subsequently, information on their age and height was gathered. Participants’ standing ear heights (vestibular height), which were used as an input parameter of motion system controller, were measured with a ruler. Experimental setup, including the six working conditions, was described to all the participants.

**Table 2:** Experimental working conditions.

	Working Condition	Value (m/s^2^)
Traction	Micro acceleration	0.28
	Slow acceleration	0.56
	Hard acceleration	0.83
Braking	Micro deceleration	−0.28
	Slow deceleration	−0.56
	Hard deceleration	−0.83

**Figure 6:**
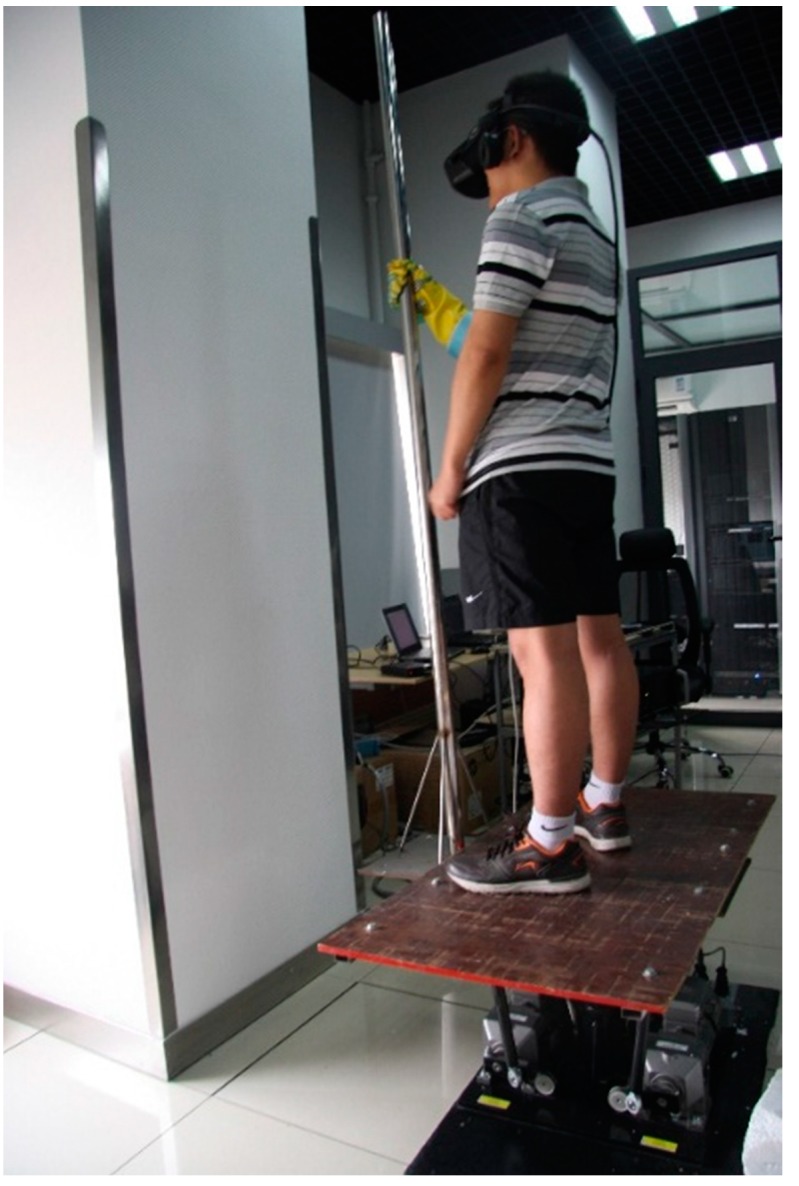
The experimental situation.

After the motion system had finished its initialization, the participant donning the head mount display stood on the platform and held the pole with his gloved hand. The experimental situation is shown in Figure 6. The experiment was conducted in three steps:

(1) Participants took part in the simulation of all the six working conditions. After the motion system finished running a set of random working conditions. Participants verbally made their judgment on which the current working condition was. Each working condition appeared at least three times.
(2) The motion system ran a simulation scenario, which was designed following the real trip between the National Library station and Weigongcun station of Beijing metro line 4, to simulate a complete running process of a train’s departure and arrival procedures. The process included three major phases: traction phase, cruise phase and braking phase. The detailed working conditions of the trip are listed in Table 3. When the simulation was running, participants continuously provided verbal judgments on which the current working condition was. Meanwhile, the grip pressure mapping system continuously recorded the grip force of participants. The sampling rate of the grip force system was set to be 5 Hz. After the simulation, a visual analog scales of motion sensation were used and participants verbally gave their ratings to the experimenter. The scales ranged from 0 to 1, with 0.5 as neutral. The motion sensation scale ranged from no fidelity (score 0) to considerable fidelity (score 1).
(3) After finishing all the above simulation tasks, each participant filled out a five-point Likert visual analog scales questionnaire to report their motion sensation of vertical direction (Z axis). The sensation scale ranged from no sensation (1), to mild (2), moderate (3), noticeable (4) and considerable sensation (5).

**Table 3:** Working conditions of the whole running process scenario.

Time (s)	Acceleration Value (m/s^2^)
0–14	0.83
14–23	0.57
23–37	0.28
37–75	0
75–83	−0.28
83–99	−0.57
99–111	−0.83

#### 2.4.2. Stage Two

The second-stage experiment was designed to investigate the correlation between hand grip force and longitudinal comfort. The preparatory work of the second-stage was similar to the one of the first-stage experiment. Acceleration value and acceleration duration of acceleration were considered as the experimental parameters in the second-stage experiment. The tested acceleration values ranged from 0.2 m/s^2^ to 1.0 m/s^2^ with 0.1 m/s^2^ as an interval, for a total of nine levels. The maximum acceleration of a rapid transit train is much lower, typically of the order of 1.0 m/s^2^ [21]. The tested acceleration duration ranged from 3 s to 10 s with 1 s as an interval, for a total of eight levels. Participants were asked to stand on the platform and hold the pole with a gloved hand. The grip pressure mapping system continuously recorded the grip force of participants. The level of acceleration value and the level of duration time were randomly chosen to avoid spurious distortions of the experimental results. Each possible combination of the acceleration value level and duration time level was presented at least two times to each participant. A visual analog scale of motion comfort was used and participants verbally reported their motion comfort at each acceleration value level and acceleration duration level. The scales range from 0 to 1 with 0.5 as neutral. The scale’s labels ranged from extremely comfortable to extremely uncomfortable.

### 2.5. Data Analysis

The pressure data was processed using Grip Research version 6.51 (which is shipped with Tekscan grip pressure mapping system) to obtain the grip force of the participants. The base grip force was different due to the individual differences between participants. Hence, the average grip force of each participant in a static state was defined as the base grip force of the participant, and incremental grip forces, which were the differences between the present grip force and the base grip force, were used to do the subsequent analysis. The mean grip force of each participant in static state, and the incremental grip forces of each participant at difference acceleration levels were calculated as follows:

(1) $$F_{ss}=\frac{1}{N_{ss\;}}\overset{N_{ss}}{\underset{n=1}{\sum }}F_{n}$$

(2) $$F_{a}=\frac{1}{N_{a\;}}\overset{N_{a}}{\underset{n=1}{\sum }}F_{n}$$

(3) $$IF_{a}=F_{a}-F_{ss}$$

where $F_{ss}$ was the average grip force in static state, $N_{ss\;}$ was the total data frame number in static state, $F_{n}$ was the grip force at data frame *n*, $F_{a}$ was the average grip force at the acceleration level of *a*, $N_{a\;}$ was the total data frame number at the acceleration level of *a*, $IF_{a}$ was the incremental grip forces at the acceleration level of *a*. Paired t-tests, Tukey’s HSD procedure and multitude regression analysis [22] were used to study the effects of incremental grip force on the longitudinal comfort score of rapid transit train. *p* < 0.05 was considered significant.

## 3. Results

### 3.1. Validity and Fidelity of the Simulation System

#### 3.1.1. Working Condition Judgment

All fifteen participants judged all the six acceleration and deceleration working condition correctly.

#### 3.1.2. Fidelity Evaluation

**Figure 7:**
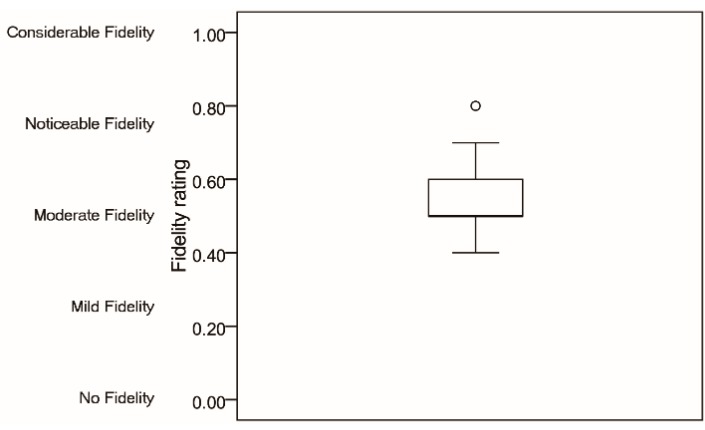
Fidelity ratings.

The fidelity ratings range is shown in Figure 7. There were 80% participants considered that the fidelity rating of the motion system was moderate or more than moderate. The average score (*M* = 0.5533, *SD* = 0.1187) indicates that the motion system had a fidelity rating between moderate and noticeable.

#### 3.1.3. Motion Sensation of Z Axis

Table 4 shows the frequency statistics of the Z axis motion sensation. The percentages of feeling no sensation, mild sensation and moderate sensation were 40.0%, 47.7% and 13.3%, respectively. There were 86.7% participants had no sensation or mild sensation on the motion along Z axis. Hence, the Z axis compensation based control algorithm could resolve the implication of the motion along Z axis.

**Table 4:** Z axis motion sensation.

	Frequency	Percentage
No sensation	6	40.0%
Mild sensation	7	46.7%
Moderate sensation	2	13.3%

The results of the present section verified the validity and fidelity of the motion system. The system was ready for the second-stage experiment.

### 3.2. Effect of Acceleration and Deceleration

**Figure 8:**
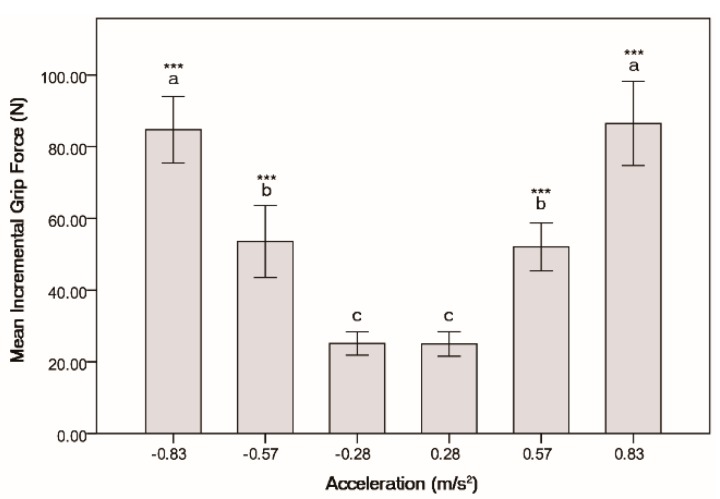
Incremental grip force at each acceleration or deceleration, *** *p* < 0.0001.

The six working conditions, shown in Table 2, could be divided into three groups according to the absolute values of the acceleration and deceleration. Paired t-tests were used to compare the grip force of each group. There was no significant different in the grip force for micro-acceleration (*M* = 24.9773, *SD* = 3.4056) and micro-deceleration (*M* = 25.1027, *SD* = 3.2506); *t*(14) = −0.135, *p* = 0.895, or for slow acceleration (*M* = 52.0513, *SD* = 6.7028) and slow deceleration (*M* = 53.5540, *SD* = 10.0233); *t*(14) = −1.027, *p* = 0.322, or for hard acceleration (*M* = 86.4893, *SD* = 11.7320) and hard deceleration (*M* = 84.7387, *SD* = 9.2731); *t*(14) = 0.794, *p* = 0.441. Tukey’s HSD showed that the incremental grip force were significantly different between each acceleration and each deceleration level; *F*(5, 84) = 169.347, *p* < 0.0001, as shown in Figure 8. The result indicated that the acceleration and deceleration values had a significant effect on the grip force, while the form of acceleration or deceleration had no significant effect on it.

### 3.3. Relationship between Acceleration and Incremental Grip Force

**Figure 9:**
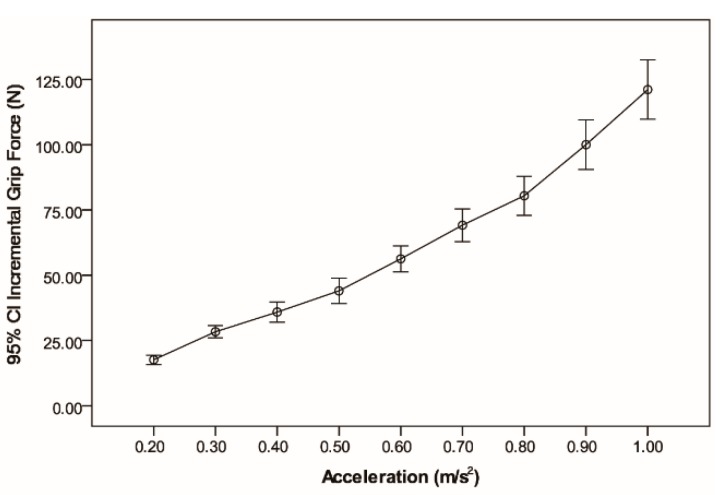
Incremental grip force at each acceleration.

There was a linear relationship between acceleration and incremental grip force (Figure 9; R-square adjusted = 0.864; *F*(1, 133) = 852.392, *p* < 0.0001). Tukey’s HSD showed that the incremental grip force were significantly different between each acceleration level; *F*(8, 126) = 126.866, *p* < 0.0001, as shown in Table 5.

**Table 5:** Incremental grip force vs. acceleration.

Acceleration	0.2	0.3	0.4	0.5	0.6	0.7	0.8	0.9	1.0
Mean incremental grip force (N)	17.636	28.377	35.931	44.041	56.306	69.189	80.467	100.02	121.12

### 3.4. Grip Force Based Comfort Prediction Model

Multiple regression analysis was used to test if the incremental grip force significantly predicted participants’ ratings of longitudinal comfort. As the results of Section 3.3, the incremental grip force and the acceleration were highly correlated, they would create multicollinearity problems. Hence, the acceleration was omitted from the regression model, the grip force and the duration were considered the predictor variables in the model.

The means, standard deviations, and intercorrelations can be found in Table 6. This combination of variables significantly predicted the comfort score, *F*(2, 1077) = 620.698, *p* < 0.0001, with both variables significantly contributing to the prediction. The beta weights, presented in Table 7, suggest that grip force contributes most to predicting the comfort score, and duration also contribute to this prediction. The adjusted *R* squared value was 0.535. This indicates that 53.5% of the variance in comfort score was explained by the model. This is a large effect.

**Table 6:** Means, standard deviations, and intercorrelations for comfort score and predictor variables (*N* = 1080).

Variable	*M*	*SD*	1	2
Score	0.6045	0.3036	0.720 ***	0.130 ***
Predictor variable				
1. GripForce	61.4543	34.3981	-	0.000
2. Duration	6.5000	2.2924		-

*** *p* < 0.0001.

**Table 7:** Simultaneous multiple regression analysis summary for grip force, duration and comfort score (*N* = 1080).

Variable	*B*	*SEB*	*β*
Grip Force	0.006	0.000	0.720 ***
Duration	0.017	0.003	0.130 ***
(Constant)	0.102	0.022	

*R^2^* = 0.535; *F*(2, 1077) = 620.698, *p* < 0.0001; *** *p* < 0.0001

The following was the regression function of the grip force, duration and longitudinal comfort:

(4) $$y\;=\;0.006x_{1}+0.017x_{2}+0.102$$

where $x_{1}$ is grip force, $x_{2}$ denotes acceleration duration, *y* is comfort score. The regression coefficient of the equation is positive, indicating that Equation (4) is an increasing function. When the grip force and acceleration duration increased, the comfort score increased, which meant that when there was more longitudinal acceleration it was more uncomfortable.

An increasing linear function will have a very large result value if the independent variables are large numbers. It would be irrational for the comfort score to be larger than 1 or less than 0. Hence, we defined the comfort score equal to 0 when the train was stopped or ran at a constant speed. When the incremental force was less than 0 or equal to 0, the comfort score value was 0. We defined the comfort score equal to 1 when the result of Equation (4) was larger than 1. The final step function of the grip force, duration and longitudinal comfort is as follows:

(5) $$y\;=\{\;\begin{array}{ll} 1 & 0.006x_{1}+0.017x_{2}+0.102>1\\ 0.006x_{1}+0.017x_{2}+0.102 & x_{1}>0\\ 0 & x_{1}\leq 0 \end{array}$$

From the function, the longitudinal comfort under any grip force and acceleration duration can be calculated.

## 4. Discussion

### 4.1. General Discussion

**Figure 10:**
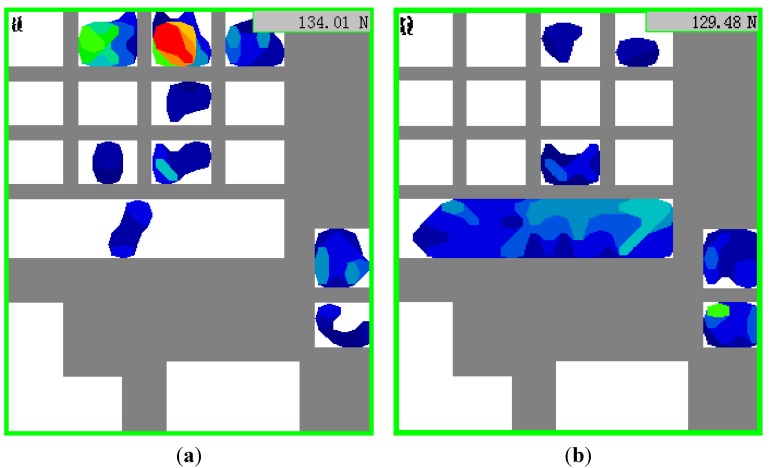
(**a**) Grip force distribution of acceleration; (**b**) Grip force distribution of deceleration.

The results of the experiment show that the incremental grip force is linearly correlated with the acceleration. The incremental grip force increased as the acceleration increased. Figure 9 shows the linear relationship between the two variables. Figure 8 shows the incremental grip force is linearly correlation with the value of acceleration but has no correlation with the direction of the acceleration vector. In the experiment, the participants stood on the standing platform facing the right side of the train and held the handrail with their right hand. Hence, when the train accelerated, more force was imposed on the fingers of the hand, and when the train decelerated, more force was imposed on the palm of the hand (see Figure 10). Although the distribution of grip force was different between acceleration and deceleration working conditions, the participants needed use the same force to overcome the same acceleration or deceleration. In the study, we analyzed the total grip force of the entire hand. Hence, the results showed that there was the no correlation between the incremental grip force and the direction of the acceleration vector.

The present results show that the incremental grip force and acceleration duration both had effects on the comfort score. The standardized coefficient shows that the incremental grip force had a greater effect on the comfort score. There was no correlation between the incremental grip force and the acceleration duration. The longitudinal comfort function was a step function. The step function ensured the value of the longitudinal comfort ranged from 0 to 1. The incremental grip force ($x_{1}$) couldn’t be infinite because it was linearly correlated with the acceleration that had a reasonable range. The function would become useless if the duration time had a large number. Actually, the distances between two adjacent stations of rapid transit system are always short (the average distance for Beijing metro line 4 is 1.2246 km), and the maximum speed of a rapid transit train is up to 80 km/h. Hence, the time duration ($x_{2}$) would not be an irrationally large value. The function was suitable for the prediction of longitudinal comfort of rapid transit trains.

### 4.2. Case Study and Comparative Analysis

#### 4.2.1. Case Study

**Figure 11:**
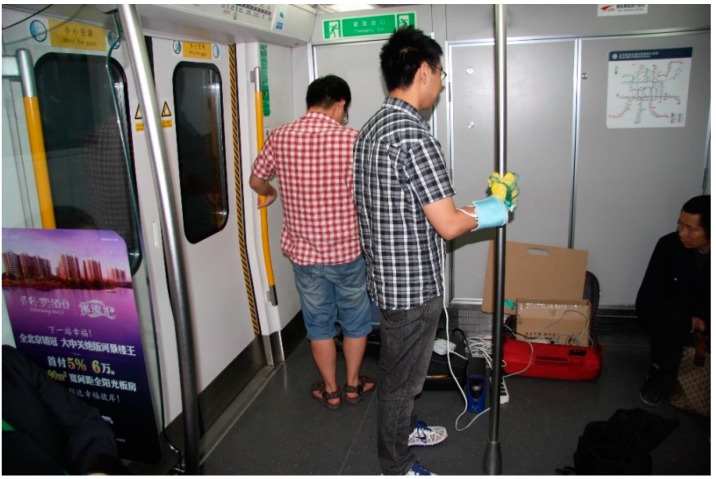
Field test situation.

A field test was conducted on a train running between National Library station and Weigongcun station of Beijing metro line 4. The working conditions of the train were shown in Table 3. A volunteer participated in the test. The grip pressure mapping system was used to gather the hand grip forces of the participant. The test situation is shown in Figure 11.

**Figure 12:**
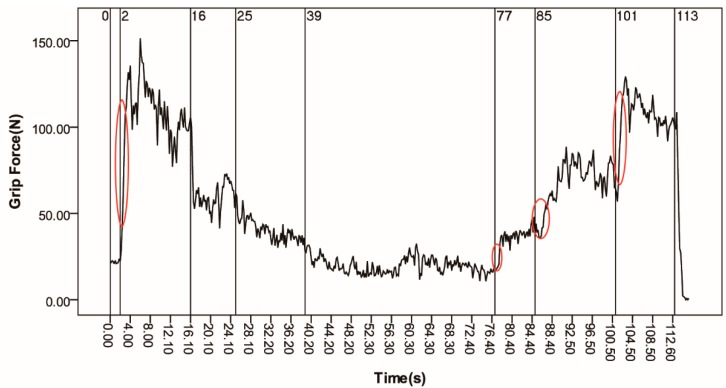
The result of hand grip force for the whole trip.

The grip force result of the participant is shown in Figure 12. The tendency of the curve approximates the working conditions. The grip force level increased or decreased when the acceleration changed. There were some steep curves, which were marked with oval tags in the figure. It should be noted that the steep curves lagged behind the change of acceleration. The steep curves correspond to the reaction of the participant. When the acceleration changed, the participant needed time to react and needed time to move [23]. Hence, the delay happened. The steep curves’ changing pattern could help us to determine the variations of working conditions.

The mean grip force (see Table 8) was calculated according to the working conditions. During the first 2 s of the trip, the train was stopped at National Library station, and the participant stood on the train and held a stanchion during the stop. The basic grip force was recorded during this time. After that, there was a traction stage from 2 s to 39 s. Subsequently, the train cruised at constant speed between 39 s and 77 s during which the grip force was also recorded as the basic grip force. At the end of the trip, there was a braking stage from 77 s to 113 s. The grip forces, incremental grip forces and the comfort scores are shown in Table 8. The incremental grip forces of traction stage and braking stage were calculated based on the mean grip force of the stopping stage and the cruising stage. The comfort scores were calculated using Equation (5).

**Table 8:** The grip forces and the comfort scores of the trip.

Working Condition	Time(s)	Mean Grip Force(N)	Incremental Grip Force (N)	Comfort Score
Stopping & cruising	0–2 & 39–77	19.7599	‒	‒
Traction	2–16	107.1465	87.3867	0.8643
	16–25	61.9873	42.2275	0.5084
	25–39	40.3354	20.5756	0.4635
Braking	77–85	35.7030	15.9432	0.3337
	85–101	68.0290	48.2691	0.6636
	101–113	105.5420	85.7822	0.8207

#### 4.2.2. Comparative Analysis

In the first-stage experiment, the grip forces of all participants were recorded when the motion system run the simulation scenario. The mean grip forces of each working condition of the simulation scenario are shown in Table 9 and Figure 8. It should be noted the working conditions in the simulation trip were the same as ones in the case study trip. The two incremental grip force were strongly correlated, *r*(6) = 0.995, *p* < 0.0001. Wilcoxon signed rank test was conducted between incremental grip force of simulation trip and case study trip for different working conditions. No significant difference was found among the pairs between the trips under any working conditions (*p* = 0.116). This means that the simulation system and the grip force based method were valid to evaluate the longitudinal comfort of rapid transit trains.

**Table 9:** Incremental grip force of simulation trip and case study trip.

Working Condition	Time (s)	Mean Incremental Grip Force (N)	
		Simulation Trip	Case Study Trip
Traction	2–16	86.4893	87.3867
	16–25	52.0513	42.2275
	25–39	24.9773	20.5756
Braking	77–85	25.1027	15.9432
	85–101	53.5540	48.2691
	101–113	84.7387	85.7822

## 5. Conclusions

In the present study, a simulation system was set up and a two-stage experiment was designed to investigate the role of the grip force on the longitudinal comfort of rapid transit trains. The results of the experiment show that the incremental grip force was linearly correlated with the acceleration value, while the incremental grip force had no correlation with the direction of the acceleration vector. The results also show that the effects of grip force and acceleration duration on the longitudinal comfort of rapid transit train were significant. Based on multiple regression analysis, a step function model was established to predict longitudinal comfort of rapid transit train using the incremental grip force and time duration. Field tests verified the feasibility and practicably of the model. Furthermore, a comparative analysis shows that the simulation system and the grip force based model were valid to support the laboratory studies on the longitudinal comfort of rapid transit trains.
